# Recruitment of Underrepresented Older Adults in Mental Health Research: A Scoping Review

**DOI:** 10.1093/geroni/igaf122.3837

**Published:** 2025-12-31

**Authors:** Maayra Butt, Vanessa Flores, Kimberly Cobos, Brenna Renn

**Affiliations:** University of Nevada, Las Vegas, Las Vegas, Nevada, United States; University of Nevada, Las Vegas, Las Vegas, Nevada, United States; University of Nevada, Las Vegas, Las Vegas, Nevada, United States; University of Nevada, Las Vegas, Las Vegas, Nevada, United States

## Abstract

Older adults, especially those identifying as multiracial or as racial or ethnic minorities, are drastically underrepresented in mental health research. Underrepresentation limits generalizability of study findings and hinders equitable mental health. This scoping review: 1) summarizes strategies to successfully recruit and retain diverse participants and 2) identifies recruitment barriers and proposed solutions. PubMed, PsycInfo, and Embase were searched from inception to March 2025. Eligible studies included Census-defined racial and ethnic minority middle-aged and older adults; focused on recruitment and retention; and addressed late-life affective disorders (depression, anxiety, and PTSD). Two independent raters assessed studies at each phase, with consensus adjudicated by the senior author. A total of 1091 papers were screened at the title/abstract stage and 325 papers were screened at the full text stage, yielding 35 papers for inclusion. Recruitment and retention strategies focused on partnering with community groups (i.e. faith based organizations), alleviating structural barriers (i.e. lack of transportation), and providing education regarding study purpose. Recruitment was sometimes hindered by mental health stigma, mistrust, eligibility requirements of a formal mental health diagnosis, and general difficulty enrolling racial and ethnic minority men. Papers underscored the importance of developing pre-recruitment community partnerships, involving community members on study design, and presenting information through discussion groups and at organizations serving minority populations. Effective recruitment of racially and ethnically diverse older adults into mental health research requires strategies that advance equity, accessibility, and community engagement. Future work should assess older adult perspectives and factors they consider when deciding to participate in research.

